# Cancer Genetic Services in a Low- to Middle-Income Country: Cross-Sectional Survey Assessing Willingness to Undergo and Pay for Germline Genetic Testing

**DOI:** 10.1200/GO.21.00140

**Published:** 2023-02-28

**Authors:** Prisca O. Adejumo, Toyin I.G. Aniagwu, Olutosin A. Awolude, Babatunde Adedokun, Makayla Kochheiser, Anthonia Sowunmi, Abiodun Popoola, Oladosu Ojengbede, Dezheng Huo, Olufunmilayo I. Olopade

**Affiliations:** ^1^ Department of Nursing, College of Medicine, University of Ibadan, Nigeria; ^2^ School of Occupational Health Nursing, University College Hospital, Ibadan, Nigeria; ^3^ Department of Obstetrics and Gynaecology, University College Hospital, Ibadan, Oyo, Nigeria; ^4^ Center for Clinical Cancer Genetics & Global Health, Department of Medicine, The University of Chicago, Chicago, IL; ^5^ Lagos State University Teaching Hospital, Lagos, Nigeria; ^6^ Center for Population and Reproductive Health, College of Medicine, University of Ibadan, Ibadan, Oyo, Nigeria; ^7^ Department of Public Health Sciences, The University of Chicago, Chicago, IL

## Abstract

Cancer genetic testing (CGT), a pathway to personalized medicine, is also being embraced in Nigeria. However, little is known about the influence of demographics and perceptions on individuals' willingness to access and pay for CGT. This study assessed patients' willingness to undergo CGT in southwest Nigeria as a catalyst for sustainable Cancer Risk Management Program.

**METHODS:**

This was a cross-sectional study using semistructured questionnaire to interview 362 patients with cancer and 10 referred first-degree relatives between July 2018 and February 2020. Participants from three Nigerian teaching hospitals—University College Hospital, Ibadan, Lagos State University Teaching Hospital, Lagos, and Lagos University Teaching Hospital, Lagos, received genetic counseling and had subsequent CGT. Primary outcomes were willingness to undergo CGT in determining cancer risk and the willingness to pay for it. Ethical approval was from appropriate ethics committees of participating hospitals. Data were analyzed with SPSS version 22. Univariate comparison of categorical variables was performed by χ^2^ test, multivariate analysis by logistic regression.

**RESULTS:**

The participants from University College Hospital (56.2%), Lagos State University Teaching Hospital (26.3%), and Lagos University Teaching Hospital (17.5%) were mostly female (98.4%). Mean age was 48.8 years ± 11.79. Three hundred twenty-two (86.6%) patients and first-degree relatives were willing to take the test, of whom 231 (71.1%) were willing to pay for it. more than half (53.6%) of the participants were willing to pay between N10,000 and N30,000, which is less than $100 US dollars. Sociodemographic variables and willingness to test showed no association (*P* > .05). Education and ethnicity were found to be associated with their willingness to pay for CGT (*P* ≤ .05).

**CONCLUSION:**

Learning clinically relevant details toward cancer prevention informs health-related decisions in patients and relatives, a motivator for willingness to pay for genetic testing in low- and middle-income countries. Increased awareness may influence outcomes of cancer risk management.

## INTRODUCTION

Breast cancer has become the leading cancer affecting women globally, particularly in low-resource countries.^1,2^ It is the most frequently diagnosed cancer in women, with 1.7 million new cases and 521,900 deaths in 2012 compared with 1.38 million new cases and 458,000 deaths in 2008,^3,4^ representing 25% of all new cancer cases and 15% of all cancer deaths among females.^2,4,5^

However, breast cancer outcomes had improved in many developed countries across the world in the past 2 decades, with incidence and mortality trends remaining relatively stable, even decreasing. On the contrary, these cannot be said about countries in Africa, Asia, Central America, and South America.^6-8^ In Nigeria, breast cancer accounts for 34.2% of total cancer deaths^9^ and, like in other countries in sub-Saharan Africa, women present at younger age, with aggressive and advanced disease of very poor prognosis.^10^ Low- and middle-income countries account for more than 80% of breast cancer–related mortalities with inadequate awareness and low screening program in place.^11^

In addition to proven methods of reducing the burden of breast cancer, genetic science has evolved and amassed massive knowledge of genes, genetic tests, and diagnostic tools for health care providers over the years.^12^ Acceptance to test for genetic mutations is a behavior predictive of a giant step toward prevention.^13^ Although the use of genetic testing (GT) for screening of familial cancer risk has been available in global medical practice for over 20 years, such referrals are near to nonexistent in most developing countries. However, the recent findings from the largest case control studies in Nigeria, replicated in Cameroon and Uganda, showed that 15% of women with breast cancer carried a susceptibility allele for inherited breast cancer.^10,14^ This provides the opportunity for leveraging on genetic-based precision prevention in developing countries. Unfortunately, access to genetic counseling is a limiting factor. Also, testing for genetic mutations comes with responsibilities on the part of the probands/consultands and relatives.^15^

In Nigeria, where basic initial genetic information is deficient, the prevalence of cancer as well as its genetic heritability continue unhindered in at-risk families because the patients and relatives do not have access to information on genetics and genetic counseling. Moreover, as promising as genetics and genetic counseling is, there is no evidence of integrated genetic counseling services in oncology care across Nigeria. Given that genetic approach to expanded carrier screening in health care setting is fairly new in most African settings, little is known about how individuals perceive its use and if they are willing to pay for the service at all.^16^ To bridge this knowledge gap, the authors developed nurse genetic counselors' curriculum for training in genetic risk assessment and provide counseling service to patients with breast cancer in three collaborating tertiary health institutions in Nigeria. Also, we developed a study on how demographics, knowledge, attitude, and practices may influence individuals' willingness to pay for cancer genetics,^13^ especially in the developing countries^17^ to test price point toward integrating genetic services in Nigeria.

Out-of-pocket cost implications on GT have been assessed in other parts of the world.^18^ Not much is known across the world, especially in an evolving genetic medicine environment like Nigeria. Even in developed and more experienced countries, cost has been established to be a barrier to effective uptake of health services in general and genetic services in particular.^19-23^ Willingness to pay for cancer genetic testing (CGT) is therefore a significant step toward prevention as it encourages definite informed decision making on genetic cancer screening^24^ since Nigerian health care users have to pay for many other services out of pocket. Generally, the Nigerian health care system has been described as not adequate in terms of accessibility, affordability, and sustainability of essential and special care services, which include oncology care.^25-27^ Women do not have access to cancer screenings as they do in much developed countries because of an array of factors, which include financing and no perceived need.^1,28^

This study aimed to provide an insight from Nigeria in determining factors associated with intention and willingness to have genetic counseling and testing among patients with breast cancer and relatives. As part of plan to establish a routine and sustainable care, the study, also ascertained the willingness of the patients to pay for CGT.

## METHODS

This cross-sectional survey assessing willingness to undergo and pay for germline GT of patients with cancer is a component of an ongoing study piloting the mainstreaming of routine GT service into clinical practice in Nigeria. The recruitment started in July 2018 at University College Hospital, Ibadan, Nigeria, and in August 2018, it was extended to Lagos University Teaching Hospital and Lagos State University Teaching Hospital, both in Lagos. At the three participating tertiary health institutions, patients with selected cancers were informed of the availability of genetic counseling and testing (GCT) services at the clinics. Patients with breast, ovarian, endometrial, and prostate cancers including first-degree relatives (FDRs) irrespective of family history of any cancer, who were receiving genetic counseling for the first time, participated in the study. They received genetic counseling at enrollment, by professionally trained nurse-genetic counselor, and informed consent was obtained.

Although this is predominantly breast cancer study, other cancers bring different flavors to the study in respect to the specificity required in the provision of genetic counseling to cancers.

In-depth genetic counseling (GC) was provided followed by an anonymous interviewer administered survey. The GC contents include taking a personal and family history with pedigree drawing; determining cancer mutation risk, educating about genetics of hereditary breast cancer, GT methods, cost, and the meaning of the results; and discussing the benefits and risks of GT, confidentiality issues, and importance of sharing the testing results with family members. A semistructured questionnaire was, thereafter, administered on the participants. Ethical approval was obtained from the appropriate ethical committees of the teaching hospitals with the ethical approval numbers UI/EC/18/0251 and LREC/06/10/1225 from Ibadan and Lagos, respectively.

Sociodemographic data and family history of cancer were captured for each participant, including family history of cancer. Perception of causes of respondents' cancer was assessed by a response of yes/no to a list of causes. Willingness of respondents to discuss cancer risk with their relatives was evaluated with four items. Likely benefits and barriers to GT and testing service were explored. Willingness to pay out of pocket for GT was evaluated with three items, which followed the exploration of their willingness to undergo the test. Participants were asked to indicate how much they were willing to pay out of pocket for GT service. Amount in naira with five choices offered were influenced by current practice by commercial laboratories in the United States, United Kingdom, and South Africa at the time of study using an equivalence of about $500 US dollars (USD) cutoff as the threshold limit of payment. Readiness to discuss the result of GT with relatives and the specific relatives to discuss it with were explored.

Data were analyzed using SPSS version 22. Univariate comparison of all categorical variables was performed by χ^2^ test and multivariate analysis. Expected outcomes include perceived causes of cancer, perceived benefits of GCT, willingness to discuss their cancer and genetic counseling and risk assessment with relatives, willingness to undergo GT, and pay for the test.

## RESULTS

A total of 372 respondents participated in the study between July 2018 and February 2020. Most (97.3%) had at least one cancer, while the remaining 2.7% were FDRs with strong family history of cancer. Respondents from University College Hospital were 56.2%, while 26.3% and 17.5% were from Lagos State University Teaching Hospital and Lagos University Teaching Hospital, respectively. Mean age of respondents was 48.8 years ± 11.79 and most (98.4%) were female. The respondents were of diverse ethnicity, and educational qualification ranged from elementary to postgraduate, with a mean monthly income of N60,545.69 87 ± N87,611.91 (Table 1).

**TABLE 1:**
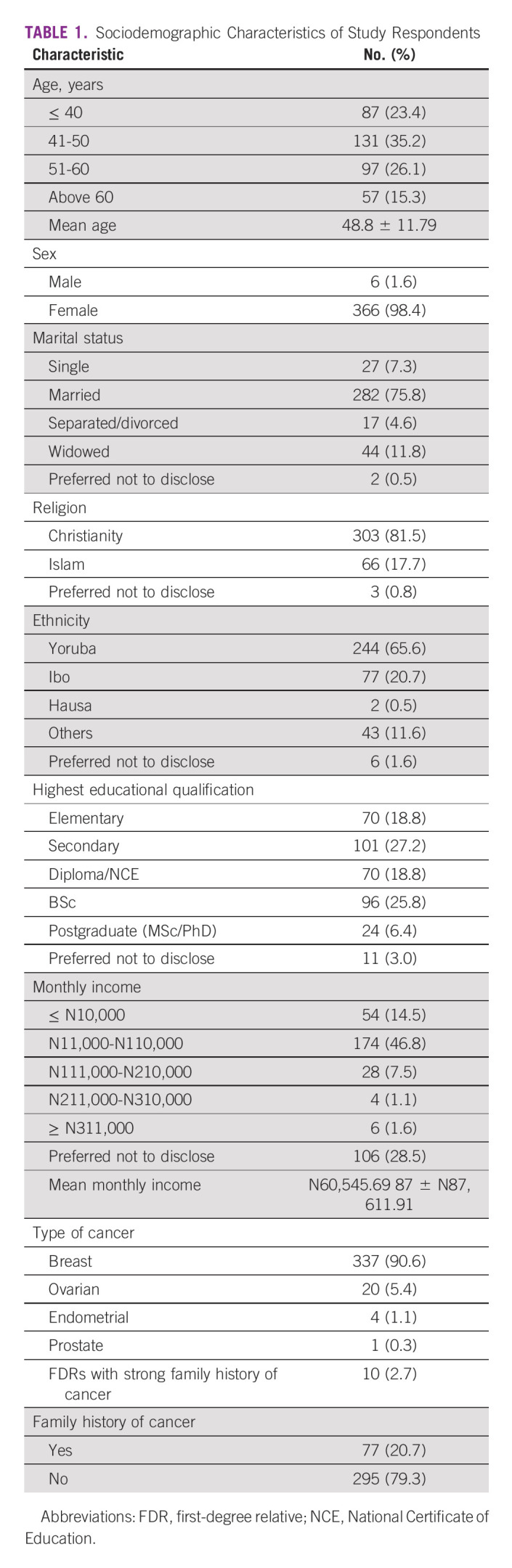
Sociodemographic Characteristics of Study Respondents

One fifth (20.7%) of the respondents reported positive family history of cancer, while there were a variety of perceived causes of the cancer by the respondents, of which stress (12.7%), heredity/family history (11.3%), unhealthy diet (9.7%), and previous history of breast cancer (9.7%) were top four, and more than a third (40.9%) could not attribute any reason for developing their cancer (Table 2). Respondents with cancer reported that they discussed their cancer with their siblings (72.0%) and all their children (64.2%). However, one fifth of the respondents discussed with both parents (20.4%), while 11.0%, 18.3%, and 45.2% of the respondents did not discuss their cancer at all with any of their close relatives (Table 3).

**TABLE 2:**
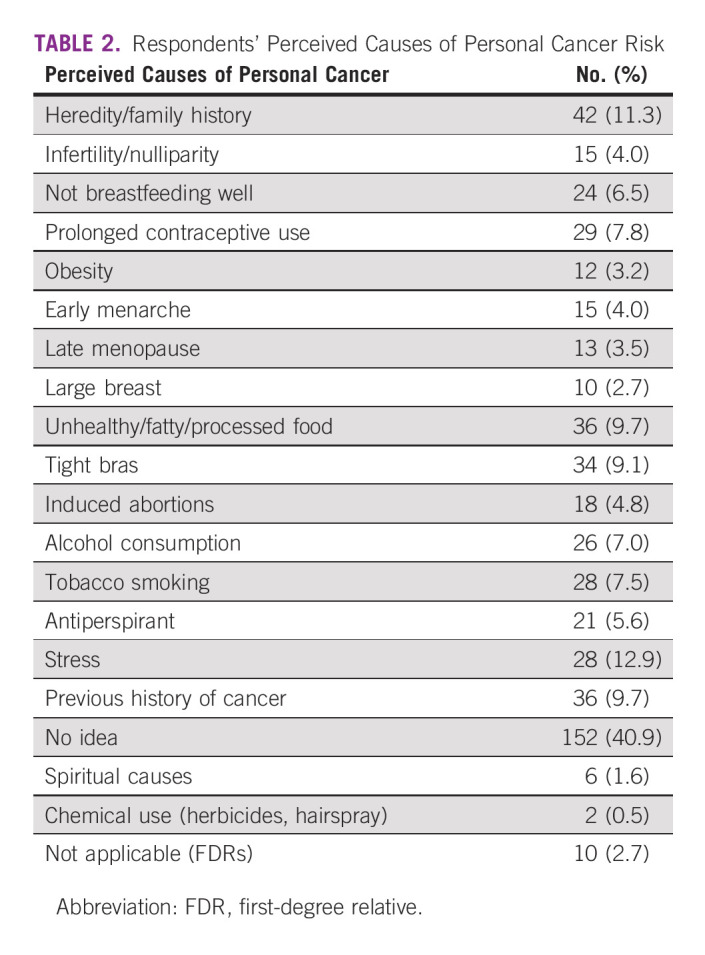
Respondents' Perceived Causes of Personal Cancer Risk

**TABLE 3:**
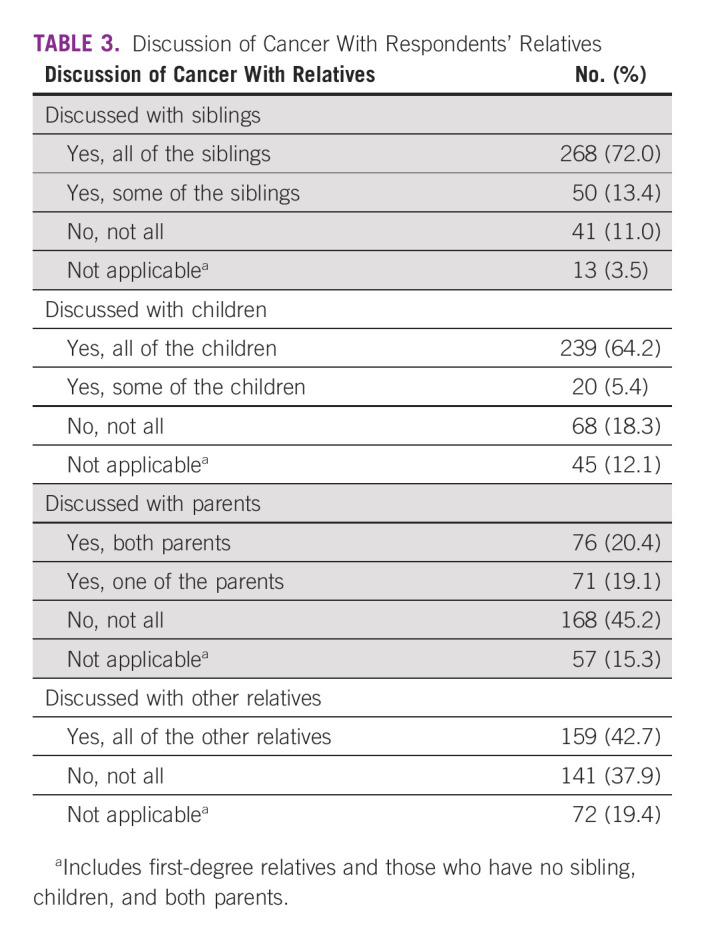
Discussion of Cancer With Respondents' Relatives

**FIG 1:**
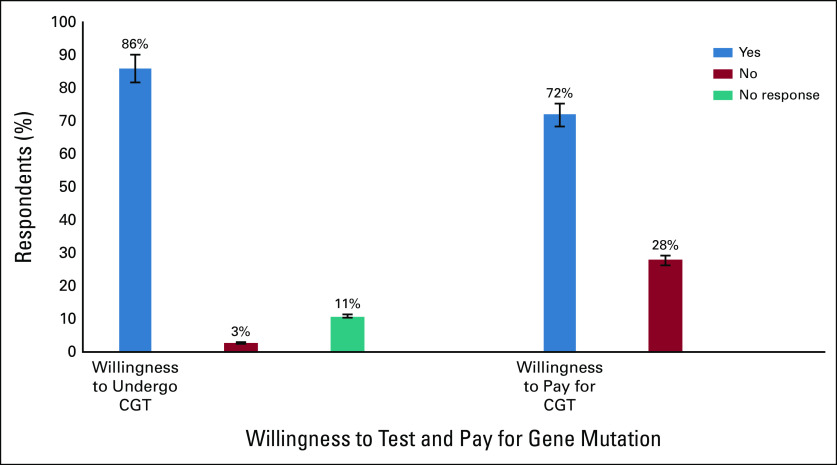
Respondents’ willingness to undergo cancer genetic test and pay for the test. CGT, cancer genetic testing.

Of the 322 participants willing to test, 231 (71.1%) were willing to pay for the test (Fig 1). Slightly more than half (53.6%) of the respondents agreed to pay between N10,000 and N30,000, which is below $100 USD, while more than a third (34.6%) were willing to pay N5,000 or less, which is less than $15 USD, and only two (0.9%) were willing to pay up to N150,000, which is about $400 USD (Table 4). However, 91 (28.2%) of the respondents who were willing to test were not ready to pay for the test. Of these, 78 (85.7%) revealed their reason to be lack of fund. Some of them added that they even had no money for treatment, while seven (7.7%) insisted that the test should be free. Few other respondents (5.5%) provided no reason for not willing to pay for GT.

**TABLE 4:**
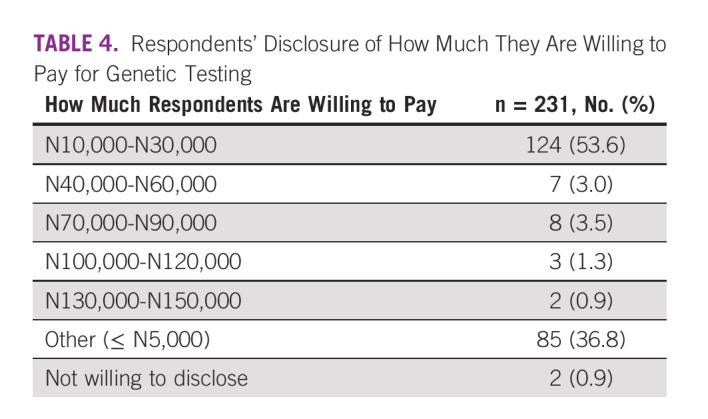
Respondents’ Disclosure of How Much They Are Willing to Pay for Genetic Testing

The findings showed that 322 (86.6%) patients and FDR were willing to undergo the test (Fig 1). Of the 10 respondents who were not willing to take the test, five (50.0%) declared that they do not want to know if they carry mutations or not, five (50.0%) stated that they do not want to be seen as bringing bad news to the family, while all the 10 (100.0%) submitted that they would be worried if they test positive.

Willingness to discuss cancer GT results with members of their family was explored among the respondents who were willing to test. Of the 319 (99.1%) who were willing to discuss the test results, top five relatives with whom they are willing to discuss their genetic test results were sisters in 239 (74.9%), daughters in 226 (70.8%), brothers in 198 (62.1%), sons in 197 (61.7%), spouses in 121 (37.9%), and mothers in 93 (29.2%; Table 5).

**TABLE 5:**
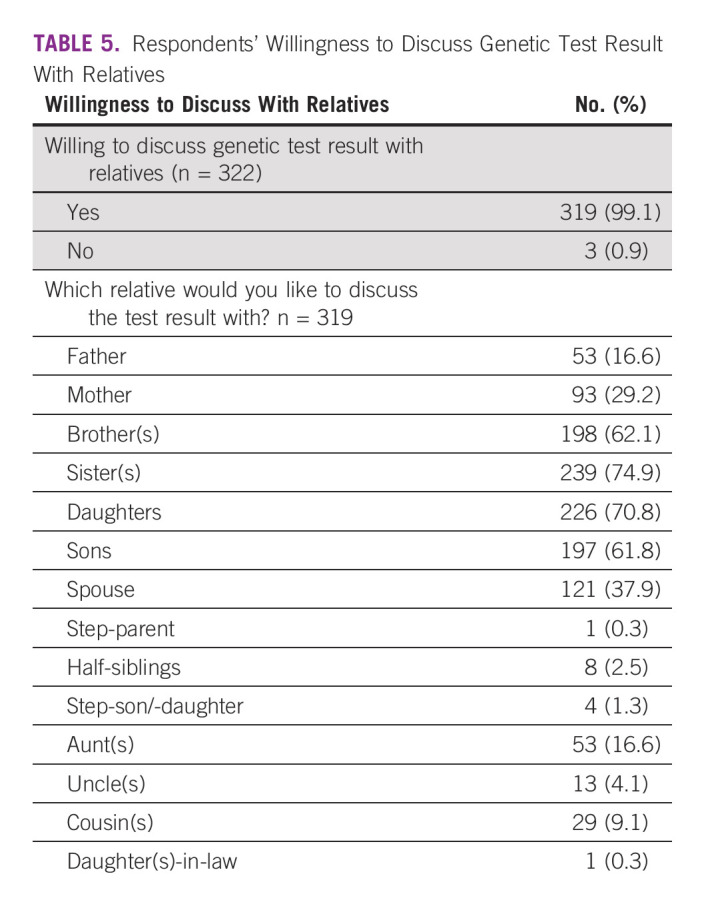
Respondents' Willingness to Discuss Genetic Test Result With Relatives

Benefits of cancer genetics and risk assessment in relatives were perceived by most patients (66.1%) to help with cancer prevention and 52.2% as early detection of cancer (Table 6), while most (65.9%) viewed cost as the major barrier to GT (Table 7).

**TABLE 6:**
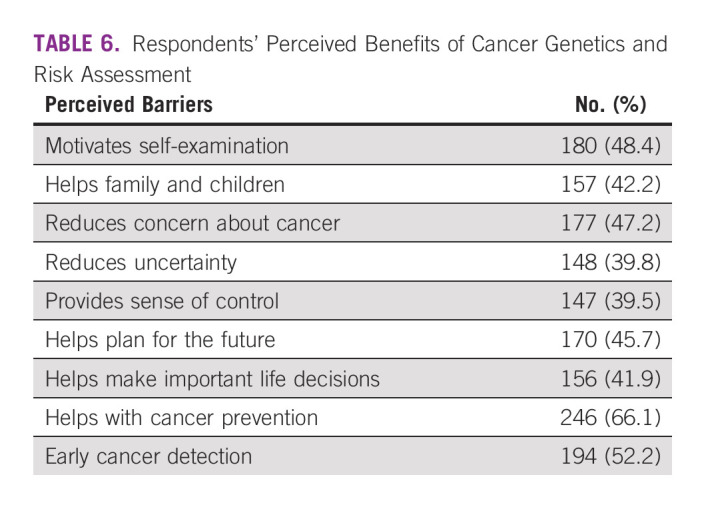
Respondents' Perceived Benefits of Cancer Genetics and Risk Assessment

**TABLE 7:**
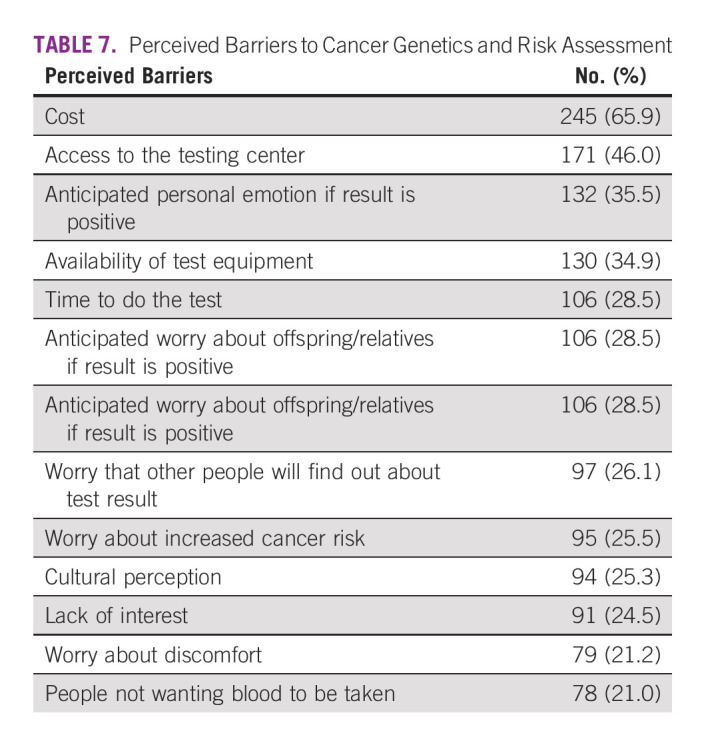
Perceived Barriers to Cancer Genetics and Risk Assessment

Bivariate analysis of the sociodemographic variables and willingness to test for genetic mutations showed no association between age, marital status of the respondents, their religion, ethnicity, educational status, and family history of cancer. However, an association was found between respondents' educational status, ethnicity, and their willingness to pay for genetic test. Other variables were not statistically significant in influencing the respondents' decision to pay for GT services (Table 8).

**TABLE 8:**
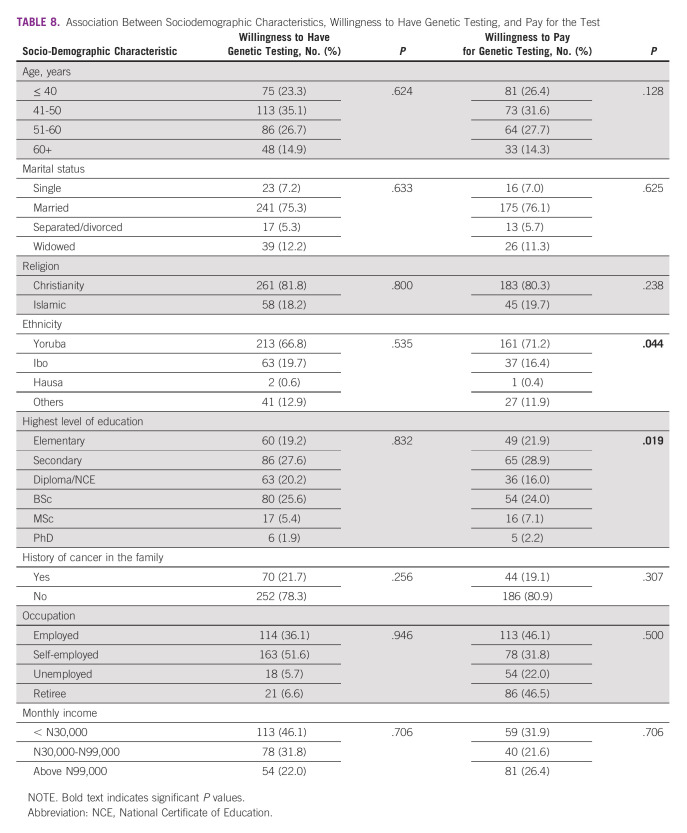
Association Between Sociodemographic Characteristics, Willingness to Have Genetic Testing, and Pay for the Test

## DISCUSSION

This study has generated data and insight into the perceptions and acceptance of genetic counseling and testing in cancer care among patients with cancer in a subsect of Nigerian population. The data also revealed the influence of social, cultural, and economic premise of the awareness and perceptions of cancer genetics and hereditary predispositions in a resource-constraint setting.

Patients with cancer and first-degree relatives in the study were found to represent the major ethnic diversities of Nigeria, and a large proportion had at least a secondary school education, which made the interaction relatively easy. Nevertheless, despite the views of possible causes of individual cancer among the patients, not all could attribute their cancer to any cause.

The proportion of respondents who linked their cancer to familial risk was not substantial with the proportion of those with family history of cancer. Such views on the basis of knowledge and beliefs may have an implication for understanding the possible influence of genetics and its influence on prevention for others in their families. Also, there was a mention of spirituality as a cause of their cancer by a few respondents, which was a factor established in previous studies.^29,30^ This reported low level of spiritual view of cancer causality may not be unrelated to the increased awareness about cancer in this setting, although late presentations still occur.^31^

Respondents indicate willingness to discuss their cancer diagnosis with their siblings and their children in their totality or part. This shows an illustration of general value of considering a patient or first-degree relative's interests as an individual encased in a cultural environment that has impression on his/her overall being.^32,33^ Many of the respondents were not willing to disclose to their parents, which is not uncommon in patients with cancer as telling loved ones about their diagnosis has been adjudged to be one of the most difficult aspects of having cancer, which leads to delay in telling those they perceived to be vulnerable and concealed their emotions from relatives to protect them.^34,35^ However, evidence has shown that talking about cancer helps people reorganize their thoughts and feelings and make sense of their experience^34^ and may provide a specific-tailored support for them.^36^

The results of willingness to test for genetic mutations among a large proportion (86.6%) of patients with cancer and FDR in this study show a significant factor toward acceptance of the test in this part of the world. Studies have found factors associated with increased acceptance and willingness, which includes risk perception and family experience^37,38^ and good knowledge of genetics.^39^ These factors, however, were not directly significant in deciding whether to have genetic test or not in this study. To encourage testing for the most at risk, women are currently advised to test for the *BRCA1/2* gene mutation if a family member has tested positive for it, or if a close relative has been diagnosed with cancer.^40^ The perceived benefits of genetic counseling and testing service for risk assessment were mostly favorable and this could be a contributory factor to their willingness to test for genetic mutations.

Most of the participants showed a readiness to discuss with at least a close member of the family, especially mothers, brothers, sisters, daughters, sons, and spouses. This becomes important for the uptake of GT^41^ and its role or implication in preventing and detecting cancer early as it is the responsibility of the proband, who is the first to be tested, to inform the relatives of the genetic test results.^42^ The postulation that male relatives are less likely to be informed of genetic test results^42^ is also seen in this study, and it has been documented that probands are more likely to share genetic test results with their children, female relatives, and relatives who they perceived had a favorable opinion about learning the results.^43-47^

Therefore, supporting probands/consultands to communicate their test results and risk information with their relatives is key to obtaining the full benefits of genetic services.^48^ Although there is a dearth of data about the reaction of relatives with whom genetic test results are shared, evidence has shown that open, positive family relationships increase the likelihood of disclosure of test results, while emotional distance, family conflict, and loss of contact decrease the likelihood of disclosure.^49-51^

The study examined the willingness to pay for genetic test among the respondents and found that although such a test is relatively new in Nigeria, most of them were willing to pay out of pocket for it. This may not be unconnected with their perception of benefits of genetic test and this singular factor has been shown in other studies to positively influence willingness to pay for genetic tests.^13,18,48^ The result did not find any association between age, marital status, level of education, monthly income, or family history of cancer and the respondents' willingness to pay for genetic test, contrary to a Canadian study in which these factors are impactful in the readiness to pay for the test.^24^

The subject of how much money respondents were willing to pay also deserves committed attention. Half of the respondents were not ready to pay more than N30,000, that is, about $100 USD, which is way below $250 USD, the cost of the current test kit used in this study. These results suggest that individuals of medium socioeconomic status are willing to pay a moderate cost for GT as payments for most health services in Nigeria are out of pocket.^49^ Interestingly, fewer respondents (2.2%) were more willing to pay between N100,000 and N150,000 ($270 USD-$400 USD) for GT out of pocket. Although this may mean a possibility for consideration of acceptability of GT among a few higher-income populations, cost was implicated by most respondents in this study to be the major barrier toward genetic counseling and testing services, as also found in previous studies.^50-53^ This willingness recorded is very significant, considering the role of decreasing socioeconomic status in health care financing.^54^

Perception of causes of cancer in probands as well as of benefits of genetic counseling and testing will awaken their interest and intention to test for deleterious mutations, which affects their care and prevention in their loved ones. Cost is a major factor in accessing genetic services and needs a holistic approach to overcome the challenges in the highly burdened health care systems of low- to middle-income countries like Nigeria and to reach the underserved populations. Culturally informed interventions and research are needed to support families in facilitating effective risk communication critical to the uptake of genetic services and the preventive impact.

In conclusion, with the recognition of the fact that 5%-10% of all cancers are hereditary, there is a growing need to improve access to GC and GT, before and after cancer diagnosis. This publication identifies opportunities to leverage on an insight from Nigeria in determining factors associated with intention to test and willingness to have genetic counseling and testing among patients with cancer and relatives. Willingness to pay for CGT identified could lead to a reform in health systems financing and exploration of other strategies for best practices in cancer care and control. The study has shown that probands have a pivotal role to play in communicating cancer risk information to family members, which promises to improve uptake of genetic services and the cancer control outlook.

## References

[1] Varughese J, Richman S: Cancer care inequity for women in resource-poor countries. Rev Obstet Gynecol 3:122-132, 2010 21364864 PMC3046761

[2] Torre LA, Bray F, Siegel RL, et al: Global cancer statistics, 2012. CA Cancer J Clin 65:87-108, 2015 25651787 10.3322/caac.21262

[3] International Agency for Research on Cancer: Globacon 2012: Estimated Cancer Incidence, Mortality and Prevalence Worldwide in 2012. http://globocan.iarc.fr/Pages/fact_sheets_cancer.aspx

[4] Ferlay J, Shin HR, Bray F, et al: Estimate of worldwide burden of cancer in 2008. Globacom 2008. Int J Cancer 127:2893-2917, 2010 21351269 10.1002/ijc.25516

[5] International Agency for Research on Cancer: Global Cancer Burden Rises to 14.1 Million New Cases in 2012: Marked Increase in Breast Cancers Must Be Addressed, No. 223, 2013

[6] Shin HR, Boniol M, Joubert C, et al: Secular trends in breast cancer mortality in five East Asian populations: Hong Kong, Japan, Korea, Singapore and Taiwan. Cancer Sci 101:1241-1246, 2010 20219071 10.1111/j.1349-7006.2010.01519.x PMC11159515

[7] Jemal A, Center MM, DeSantis C, et al: Global patterns of cancer incidence and mortality rates and trends. Cancer Epidemiol Biomark Prev 19:1893-1907, 2010 10.1158/1055-9965.EPI-10-0437 20647400

[8] Youlden DR, Cramb SM, Dunn NAM, et al: The descriptive epidemiology of female breast cancer: An international comparison of screening, incidence, survival and mortality. Cancer Epidemiol 36:237-248, 2012 22459198 10.1016/j.canep.2012.02.007

[9] World Health Organization: Cancer Country Profiles. Nigeria. 2014. http://www.who.int/cancer/country-profiles/nga_en.pdf?ua=1

[10] Zheng Y, Walsh T, Gulsuner S: Inherited breast cancer in Nigerian women. J Clin Oncol 36:2820-2825, 2018 30130155 10.1200/JCO.2018.78.3977 PMC6161833

[11] Dey S: Preventing breast cancer in LMICs via screening and/or early detection: The real and the surreal. World J Clin Oncol 5:509-519, 2014 25114864 10.5306/wjco.v5.i3.509 PMC4127620

[12] Choi J, Kim H: Effectiveness of the interventions utilized in genetic counseling. Adv Nurs 2014:725968, 2014

[13] Hann KEJ, Freeman M, Fraser L, et al: Awareness, knowledge, perceptions, and attitudes towards genetic testing for cancer risk among ethnic minority groups: A systematic review. BMC Public Health 17:503, 2017 28545429 10.1186/s12889-017-4375-8 PMC5445407

[14] Adedokun B, Zheng Y, Ndom P: Prevalence of inherited mutations in breast cancer predisposition genes among Uganda and Cameroon. Cancer Epidemiol Biomarkers Prev 29:359-367, 2020 31871109 10.1158/1055-9965.EPI-19-0506 PMC7007381

[15] Katapodi MC, Northhouse L, Piere P, et al: Hereditary breast and ovarian cancer and their at-risk relatives who did not. Oncol Nurs Forum 38:572-581, 2011 21875844 10.1188/11.ONF.572-581

[16] Clarke EV, Schneider JL, Lynch F, et al: Assessment of willingness to pay for expanded carrier screening among women and couples undergoing preconception carrier screening. PLoS One 13:e0200139, 2018 30020962 10.1371/journal.pone.0200139 PMC6051630

[17] Ngene SO, Adedokun B, Adejumo P, et al: Breast cancer genetics knowledge and testing intentions among Nigerian professional women. J Genet Couns 27:863-873, 2018 29260484 10.1007/s10897-017-0194-4

[18] Matro JM, Ruth KJ, Wong YN: Cost sharing and hereditary cancer risk: Predictors of willingness-to-pay for genetic testing. J Genet Couns 23:1002-1011, 2014 24794065 10.1007/s10897-014-9724-5 PMC4420173

[19] Han W: Health care system reforms in developing countries. J Public Health Res 1:199-207, 2012 25170464 10.4081/jphr.2012.e31 PMC4140377

[20] Kayombo EJ, Uiso FC, Mahunnah RL: Experience on healthcare utilization in seven administrative regions of Tanzania. J Ethnobiol Ethnomed 8:5, 2012 22284539 10.1186/1746-4269-8-5 PMC3277466

[21] Garcia-Subirats I, Vargas I, Mogollón-Pérez AS, et al: Barriers in access to healthcare in countries with different health systems. A cross-sectional study in municipalities of central Colombia and north-eastern Brazil. Soc Sci Med 106:204-213, 2014 24576647 10.1016/j.socscimed.2014.01.054

[22] Adedini SA, Odimegwu C, Bamiwuye O, et al: Barriers to accessing health care in Nigeria: Implications for child survival. Glob Health Action 7:23499, 2014 24647128 10.3402/gha.v7.23499 PMC3957799

[23] Aaltonen K, Miettinen J, Airio I: Cost-related barriers to use of health services and prescription medicines in Finland: A cross-sectional survey. Eur J Public Health 25:368-372, 2015 25395397 10.1093/eurpub/cku176

[24] Blouin-Bougie J, Amara N, Bouchard K, et al: Disentangling the determinants of interest and willingness-to-pay for breast cancer susceptibility testing in the general population: A cross-sectional web-based survey among women of Québec (Canada). BMJ Open 8:e016662, 2018 10.1136/bmjopen-2017-016662 PMC5855474 29487071

[25] Ezekwesili-Ofili JO, Okaka ANC: Herbal medicines in African traditional medicine, in Builders PF (ed): Herbal Medicine. London, United Kingdom, IntechOpen, 2019

[26] Oluwadare CT, Olorunfemi AA, Atiba AS, et al: A comparative assessment of access to healthcare of people living in Ekiti and Kogi States of Nigeria. Asian J Med Health 19:7-17, 2021

[27] Asakitikpi AE: Healthcare Coverage and Affordability in Nigeria: An Alternative Model to Equitable Healthcare Delivery, Universal Health Coverage, Aida Isabel Tavares. London, United Kingdom, IntechOpen, 2019

[28] Olasehinde O, Alatise OI, Arowolo OA, et al: Barriers to mammography screening in Nigeria: A survey of two communities with different access to screening facilities. Eur J Cancer Care (Engl) 28:e12986, 2019 30614109 10.1111/ecc.12986 PMC6430195

[29] Olasehinde O: 2017 Breast Cancer Screening in Nigeria: Evaluating Practices, Barriers, and Prospects. Thesis Presented to the Faculty of the Weill Cornell Graduate School of Medical Sciences. https://hdl.handle.net/1813/64744

[30] Adejumo P, Aniagwu T, Oluwatosin A, et al: Knowledge of genetic counseling among patients with breast cancer and their relatives at a Nigerian teaching hospital. J Glob Oncol 4:1-8, 2018 10.1200/JGO.17.00158 PMC6223535 30084716

[31] Olayide AS, Halimat AJ, Samuel OA, et al: Level of awareness and knowledge of breast cancer in Nigeria. A systematic review. Ethiop J Health Sci 27:163-174, 2017 28579712 10.4314/ejhs.v27i2.9 PMC5440831

[32] Denny CC, Wilfond BS, Peters JA, et al: All in the family: Disclosure of “unwanted” information to an adolescent to benefit a relative. Am J Med Genet A 146A:2719-2724, 2008 18831063 10.1002/ajmg.a.32362 PMC3143002

[33] Ghoshal A, Salins N, Damani A, et al: To tell or not to tell: Exploring the preferences and attitudes of patients and family caregivers on disclosure of a cancer-related diagnosis and prognosis. J Glob Oncol 5:1-12, 2019 10.1200/JGO.19.00132 PMC6882506 31770048

[34] Hilton S, Emslie C, Hunt K, et al: Disclosing a cancer diagnosis to friends and family: A gendered analysis of young men's and women's experiences. Qual Health Res 19:744-754, 2009 19342703 10.1177/1049732309334737 PMC2715137

[35] Badihian S, Choi EK, Kim IR, et al: Attitudes toward cancer and cancer patients in an urban Iranian population. Oncologist 22:944-950, 2017 28559414 10.1634/theoncologist.2017-0073 PMC5553964

[36] Laryionava K, Pfeil TA, Dietrich M, et al: The second patient? Family members of cancer patients and their role in end-of-life decision making. BMC Palliat Care 17:29, 2018 29454337 10.1186/s12904-018-0288-2 PMC5816525

[37] Kinney AY, Simonsen SE, Baty BJ, et al: Acceptance of genetic testing for hereditary breast ovarian cancer among study enrollees from an African American kindred. Am J Med Genet A 140:813-826, 2006 16523520 10.1002/ajmg.a.31162 PMC2562369

[38] Miron-Shatz T, Hanoch Y, Katz BA, et al: Willingness to test for BRCA1/2 in high risk women: Influenced by risk perception and family experience, rather than by objective or subjective numeracy? Judgment Decis Making 10:386-399, 2015

[39] Ewing AT, Kalu N, Cain G, et al: Factors associated with willingness to provide biospecimens for genetics research among African American cancer survivors. J Community Genet 10:471-480, 2019 30877487 10.1007/s12687-019-00411-0 PMC6754482

[40] Moyer VA: Risk assessment, genetic counseling, and genetic testing for BRCA-related cancer in women: US Preventive Services Task Force recommendation statement. Ann Intern Med 160:271-281, 2014 24366376 10.7326/M13-2747

[41] Ricker CN, Koff RB, Qu C, et al: Patient communication of cancer genetic test results in a diverse population. Transl Behav Med 8:85-94, 2018 29385580 10.1093/tbm/ibx010 PMC6065549

[42] Daly MB, Montgomery S, Bingler R, et al: Communicating genetic test results within the family: Is it lost in translation? A survey of relatives in the randomized six-step study. Fam Cancer 15:697-706, 2016 26897130 10.1007/s10689-016-9889-1 PMC5010833

[43] Montgomery SV, Barsevick AM, Egleston BL, et al: Preparing individuals to communicate genetic test results to their relatives: Report of a randomized control trial. Fam Cancer 12:537-546, 2013 23420550 10.1007/s10689-013-9609-z PMC3706561

[44] Mendes Á, Paneque M, Sousa L, et al: How communication of genetic information within the family is addressed in genetic counselling: A systematic review of research evidence. Eur J Hum Genet 24:315-325, 2016 26264439 10.1038/ejhg.2015.174 PMC4755382

[45] Blandy C, Chabal F, Stoppa-Lyonnet D, et al: Testing participation in BRCA1/2-positive families: Initiator role of index cases. Genet Test 7:225-233, 2003 14658447 10.1089/109065703322537241

[46] Dancyger C, Wiesman M, Jacobs C, et al: Communicating BRCA1/2 genetic test results within the family: A qualitative analysis. Psychol Heath 26:1018-1035, 2011 10.1080/08870446.2010.525640 21797732

[47] Fehniger J, Lin F, Beattie MS, et al: Family communication of BRCA1/2 results and family uptake of BRCA1/2 testing in a diverse population of BRCA1/2 carriers. J Genet Couns 22:603-612, 2013 23666114 10.1007/s10897-013-9592-4

[48] Wong XY, Groothuis-Oudshoorn CGM, Tan CS, et al: Women's preferences, willingness-to-pay, and predicted uptake for single-nucleotide polymorphism gene testing to guide personalized breast cancer screening strategies: A discrete choice experiment. Patient Prefer Adherence 12:1837-1852, 2018 30271127 10.2147/PPA.S171348 PMC6154732

[49] Aregbeshola BS, Khan SM: Out-of-pocket payments, catastrophic health expenditure and poverty among households in Nigeria 2010. Int J Health Policy Manag 7:798-806, 2018 30316228 10.15171/ijhpm.2018.19 PMC6186489

[50] Anderson B, McLosky J, Wasilevich E, et al: Barriers and facilitators for utilization of genetic counseling and risk assessment services in young female breast cancer survivors. J Cancer Epidemiol 2012:298745, 2012 23150731 10.1155/2012/298745 PMC3485517

[51] Steffen LE, Du R, Gammon A, et al: Genetic testing in a population-based sample of breast and ovarian cancer survivors from the REACH randomized trial: Cost barriers and moderators of counseling mode. Cancer Epidemiol Biomarkers Prev 26:1772-1780, 2017 28971986 10.1158/1055-9965.EPI-17-0389 PMC5712253

[52] Swink A, Nair A, Hoof P, et al: Barriers to the utilization of genetic testing and genetic counseling in patients with suspected hereditary breast and ovarian cancers. Proc (Bayl Univ Med Cent) 32:340-344, 2019 31384183 10.1080/08998280.2019.1612702 PMC6650213

[53] Fogleman AJ, Zahnd WE, Lipka AE, et al: Knowledge, attitudes, and perceived barriers towards genetic testing across three rural Illinois communities. J Community Genet 10:417-423, 2019 30673953 10.1007/s12687-019-00407-w PMC6591342

[54] Onwujekwe OE, Uzochukwu BS, Obikeze EN, et al: Investigating determinants of out-of-pocket spending and strategies for coping with payments for healthcare in southeast Nigeria. BMC Health Serv Res 10:67, 2010 20233454 10.1186/1472-6963-10-67 PMC2851710

